# Supplementary material for: Morphological Analysis of Poly(4,4′-oxydiphenylene-pyromellitimide)-Based Organic Solvent Nanofiltration Membranes Formed by the Solution Method
Source: Membranes (Basel). 2022 Dec 7;12(12):1235. doi: 10.3390/membranes12121235 (PMC9782061; doi:10.3390/membranes12121235)
Supplement: Supplementary file 1 [file membranes-12-01235-s001.zip › membranes-2048481-supplementary.pdf]

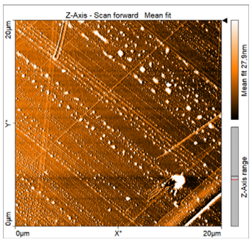

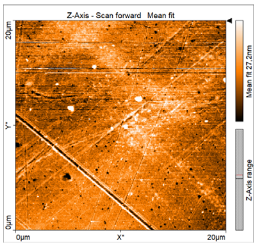

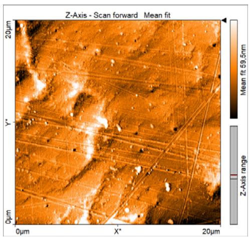

a - 170 °C **b - 200 °C** **c - 250 °C**

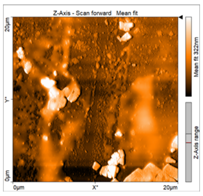

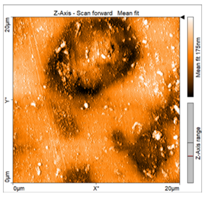

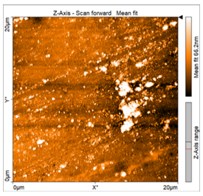

d - 300 °C **e - 350 °C** **f - 370 °C**

Figure S1. AFM images of free surface of PUI copolymer at different stages of warming (a) -170°C, (b)-200°C, (c)-250°C, (d)-300°C, (e)- 350°C (f)-370°C, in topography mode; the scanning matrix is 20 × 20 μm.

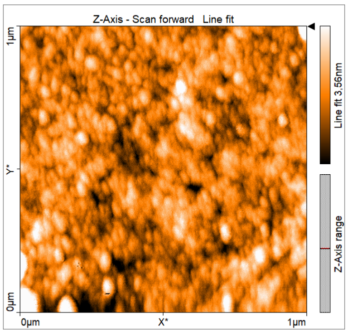

a

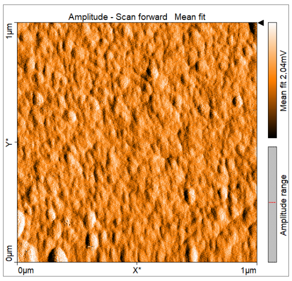

b

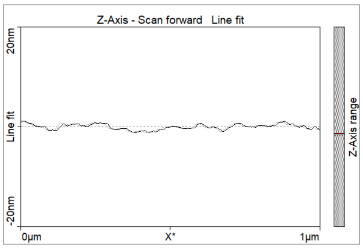

Ra = 12.39 nm

Rq = 14.69 nm

c

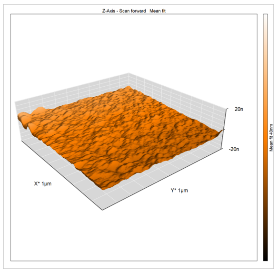

d

Figure S2. AFM images of free surface of PUI copolymer film heated at °C, in topography mode (a), amplitudes (*b*), profile of the selected surface area (*c*), 3D-image (*d*).

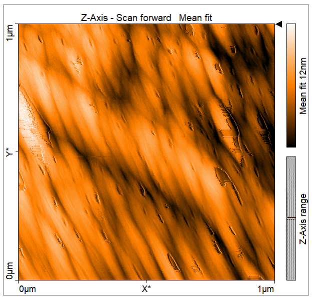

a

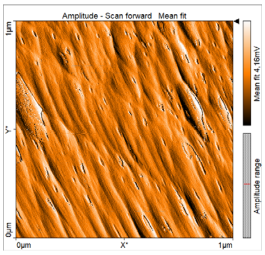

b

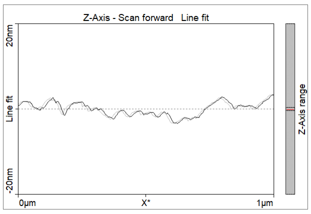

Ra = 49.23 nm

Rq = 57.70 nm

c

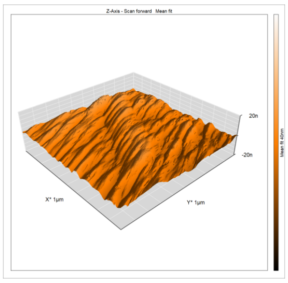

d

Figure S3. AFM images of free surface of PUI copolymer film heated at 250°C(sample No. 1), in topography mode (a), amplitudes (*b*), profile of the selected surface area *(c)*, 3D-image *(d)*.

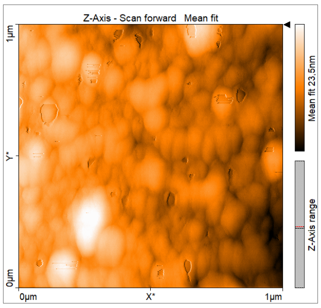

a

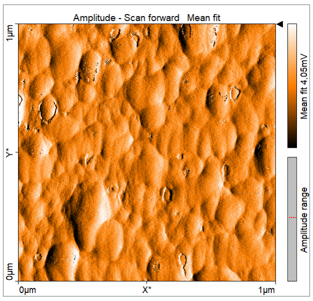

b

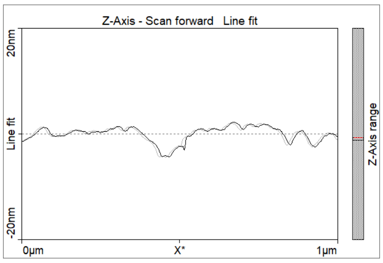

Ra = 11.60 nm

Rq = 18.05 nm

c

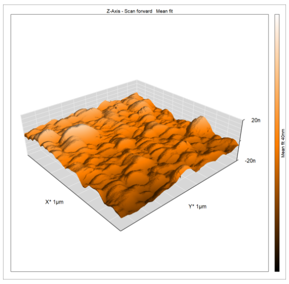

d

Figure S4. AFM images of free surface of PUI copolymer film heated at °C (sample No. 1), in topography mode (a), amplitudes (*b*), profile of the selected surface area *(c)*, 3D-image *(d)*.

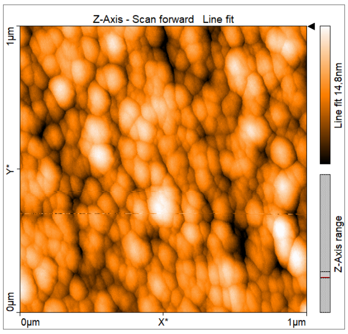

a

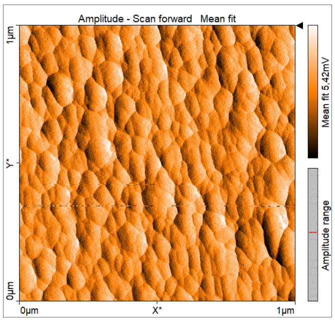

b

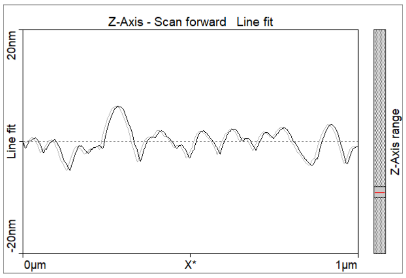

Ra = 130.18 nm

Rq = 150.85 nm

c

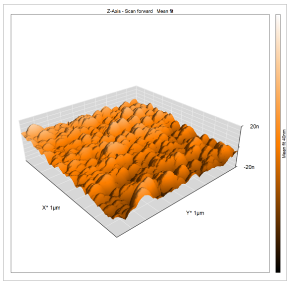

d

Figure S5. AFM images of free surface of PUI copolymer film heated at 350°C (sample No. 1), in topography mode (a), amplitudes (*b*), profile of the selected surface area *(c)*, 3D-image *(d)*.

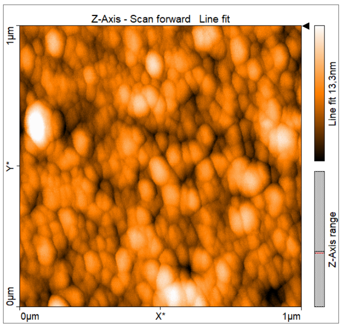

a

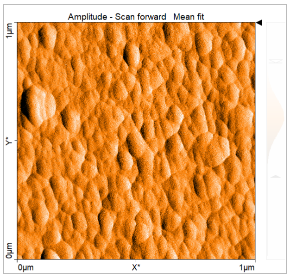

b

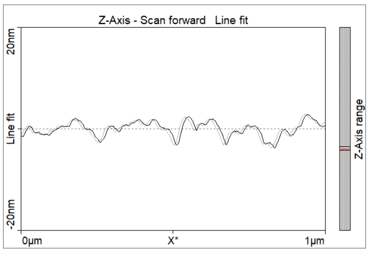

Ra = 46.85 nm

Rq = 55.19 nm

c

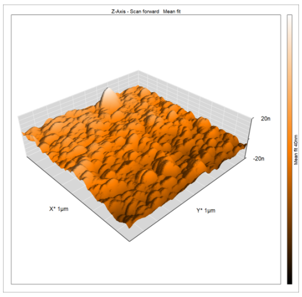

d

Figure S6. AFM images of free surface of PUI copolymer film heated at 370°C (sample No. 1), in topography mode (a), amplitudes (*b*), profile of the selected surface area *(c)*, 3D-image *(d)*.

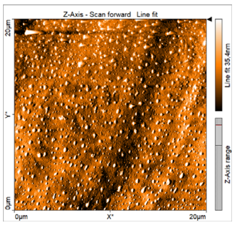

a - 170 °C

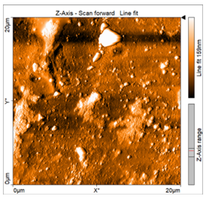

b - 200 °C

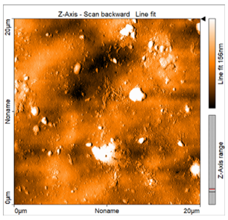

c - 250 °C

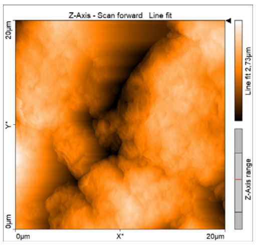

d - 300° C

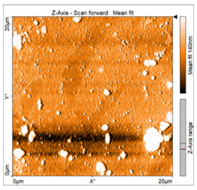

e - 350° C

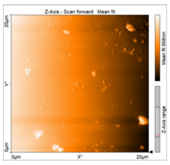

f - 370° C

Figure S7. AFM images of free surface of ***PUI2***copolymer at different stages of warming (a) -170°C, (b)-200°C, (c)-250°C, (d)-300°C, (e)- 350°C (f)-370°C, in topography mode; the scanning matrix is 20 × 20 µm.

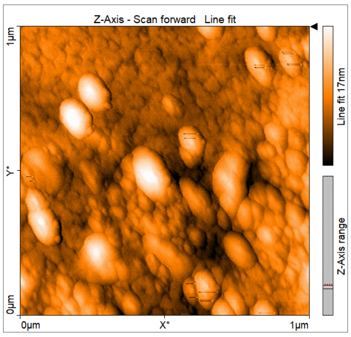

a

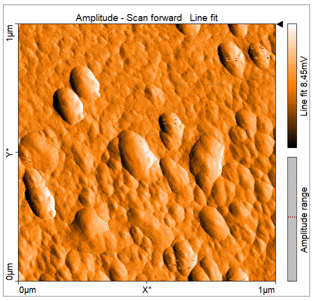

b

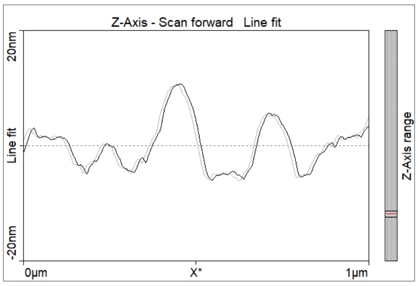

Ra = 85.65 nm

Rq = 99.42 nm

c

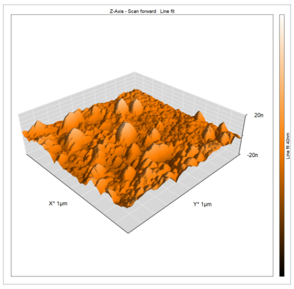

d

Figure S8. AFM images of free surface of ***PUI2*** copolymer film heated at 250°C(sample No. 2), in topography mode (a), amplitudes (*b*), profile of the selected surface area *(c)*, 3D-image *(d)*.

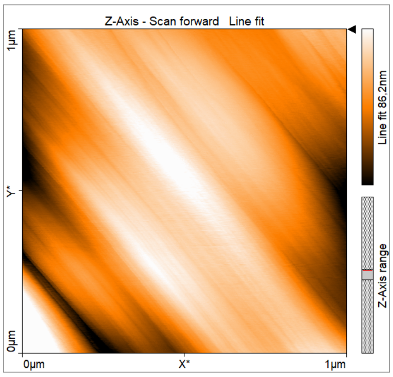

a

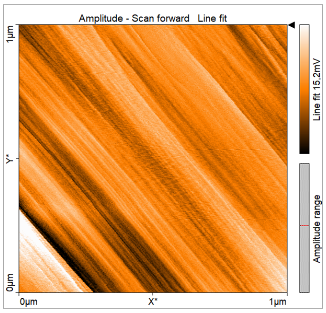

b

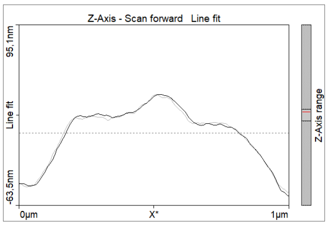

Ra = 140.44 nm

Rq = 167.54 nm

c

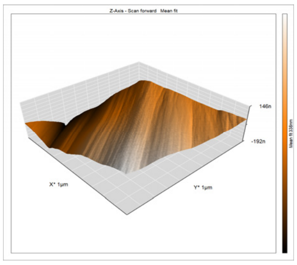

d

Figure S9. AFM images of free surface of ***PUI2*** copolymer film heated at 300°C(sample No. 2), in topography mode (a), amplitudes (*b*), profile of the selected surface area *(c)*, 3D-image *(d)*.

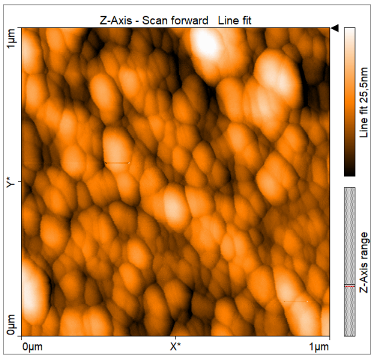

a

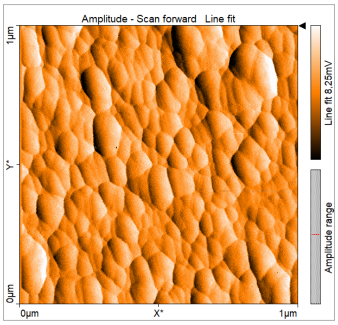

b

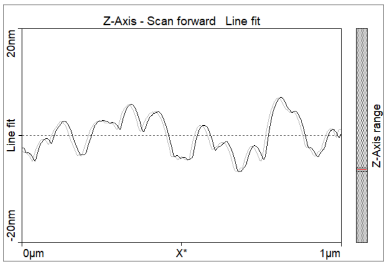

Ra = 42.56 nm

Rq = 48.31 nm

c

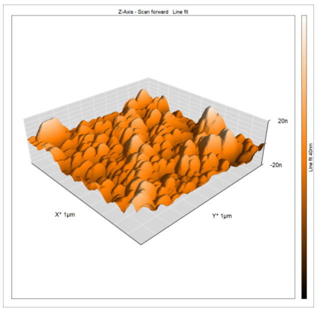

d

Figure S10. AFM images of free surface of ***PUI2*** copolymer film heated at 350°C(sample No. 2), in topography mode (a), amplitudes (*b*), profile of the selected surface area *(c)*, 3D-image *(d)*.

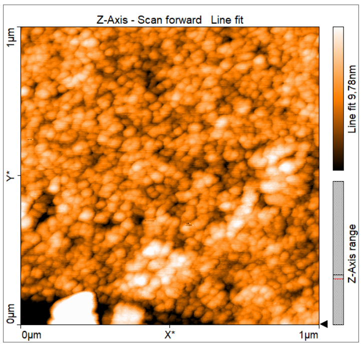

a

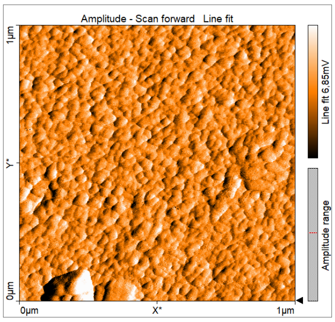

b

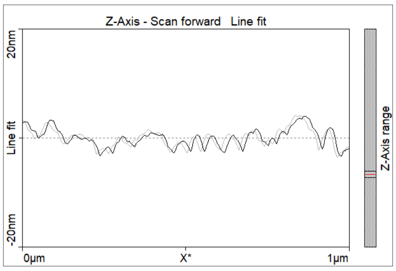

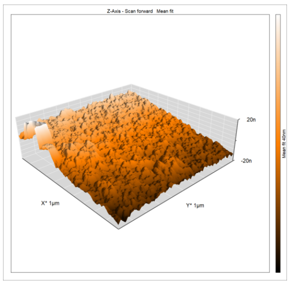

Ra = 75.96 nm

Rq = 92.90 nm

c

d

Figure S11. AFM images of free surface of ***PUI2*** copolymer film heated at 370°C(sample No. 2), in topography mode (a), amplitudes *(b)*, profile of the selected surface area *(c)*, 3D-image *(d)*.

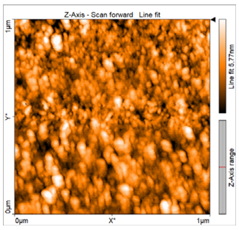

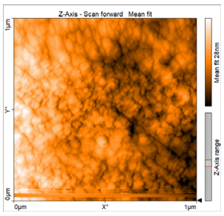

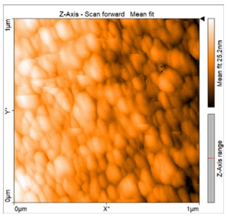

a

b

c

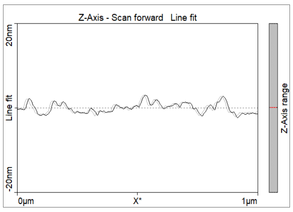

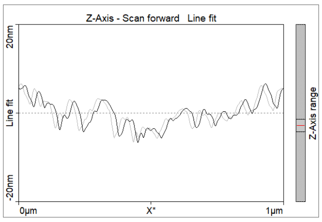

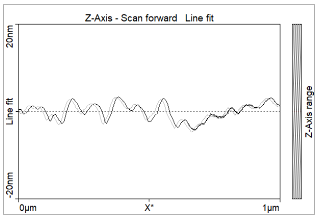

d

e

f

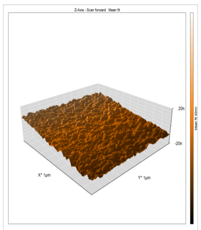

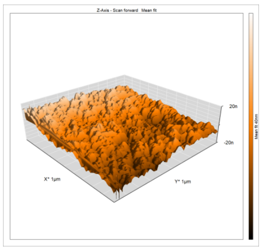

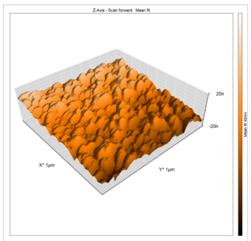

Ra = 9.699 nm

Rq = 11.349 nm

Ra = 20.56 nm

Rq = 23.01 nm

Ra = 4.39 nm

Rq = 5.177 nm

g **h** **i**

Figure S12. AFM images of the free surface of the ***PUI*** polymer film subjected to hydrolysis by various mixtures after heating: topography (a), profile of the selected surface area (d), 3D-image (g), hydrolysis by composition I (a mixture of hydrochloric and acetic acids in a ratio of 10:90); topography (b), profile of the selected surface area (e), 3D-image (h), hydrolysis by composition II (a mixture of hydrochloric and acetic acids in a ratio of 90:10); topography (c), profile of the selected surface area (f), 3D-image (i), hydrolysis by composition III (a mixture of CF_3COOH + H_2O in a ratio of 50:50).

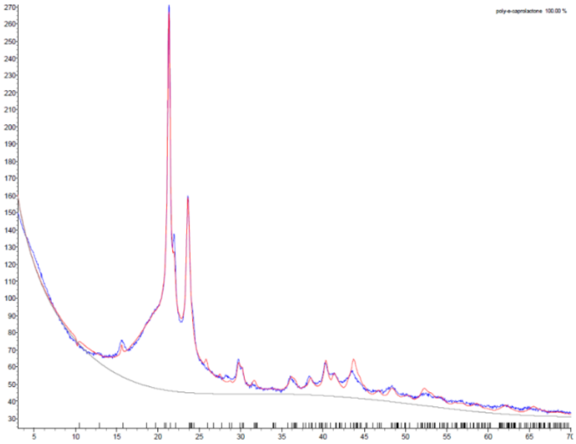

Figure S13. Polycaprolactone diffractogram.

IR studies of thermalized films.

The polyurethaneimide films from which the membranes were formed were examined by infrared spectroscopy. The IR spectrum of poly(urethane-imides) thermalized at various temperatures (170°C, 300°C, 350 °C) is shown in Figure 14 valence N-H vibrations of the urethane fragment are found in the range of wave numbers: 3310-3450 cm-1, a slight broadening of which is probably due to the participation of N-H in formation of hydrogen bonds. The bands having low intensity and found in the range of 3040-3090 cm-1 can be attributed to valence C-H vibrations of aromatic fragments of diamines and dianhydrides. While for aliphatic fragments, bands of valence C-H oscillations were found in the region of 2870-2990 cm-1. The presence of the imide cycle is indicated by the presence of several bands: low intensity bands at 1777 and high intensity bands at 1721 cm-1, which are usually attributed to asymmetric and symmetric C = O oscillations of the imide cycle. The band of symmetrical C = O vibrations of the imide cycle partially overlaps with the bands of C = O vibrations of the caprolactone and urethane fragments, the latter of which manifests itself as a shoulder at 1620 cm-1 and this indicates its participation in the formation of hydrogen bonds.

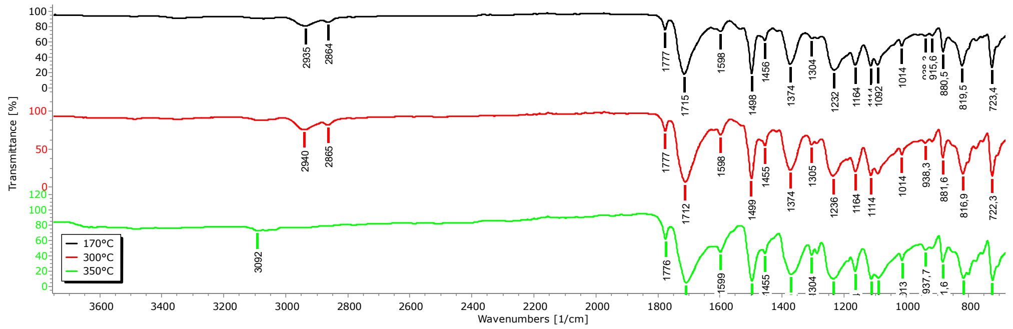

Figure S14. IR spectrum of polyurethane-imide film thermalized at 170°C, 300°C and 350°C.

As a result of thermolysis of poly (urethane imide) at high temperatures (350°C), a partial destruction of the aliphatic block occurs, which manifests itself in the IR spectra of thermalized samples in the form of a change in the intensity of a number of their bands. Namely, there are no bands in the region of 2941 and 2865 cm-1 and corresponding to the C-H fluctuations of aliphatic groups. It should also be noted a significant decrease in the intensity of the bands with = Iso vibrations of the urethane fragment at 1620 and 1164 cm-1, respectively.
